# The Impact of Sequential Organ Failure Assessment (SOFA) Score on Mortality in Geriatric Patients With Sepsis and Septic Shock in the ICU

**DOI:** 10.7759/cureus.30887

**Published:** 2022-10-30

**Authors:** Hilal Sipahioglu, Sami Bahcebası

**Affiliations:** 1 Intensive Care Unit, Kayseri City Training and Research Hospital, Kayseri, TUR; 2 Internal Medicine, Kayseri City Training and Research Hospital, Kayseri, TUR

**Keywords:** albumin, sofa, mortality, geriatric, sepsis

## Abstract

Objective

One of the most common causes of mortality and morbidity in elderly patients is sepsis. Malnutrition is widespread in elderly patients, affecting mortality and morbidity. The present study aimed to evaluate the clinical features of patients hospitalized in the tertiary intensive care unit with the diagnosis of sepsis, as well as the effects of the Sequential Organ Failure Assessment (SOFA) score, prealbumin, albumin, and other laboratory parameters on hospital mortality.

Methods

The patients were divided into two groups according to their survival. The demographic and clinical characteristics of the two groups were compared. Independent risk factors affecting mortality were determined by logistic regression.

Results

A total of 653 patients admitted to the medical ICU were evaluated out of which 254 geriatric patients with sepsis and septic shock were included. There was in-hospital mortality in 122 (48%) patients. There was no difference in age in both groups (76 (71-84) vs. 76 (70-84), p=0.896). BUN (p=0.013), LDH (p=0.014), LDH/albumin (p<0.001), BUN/albumin (p<0.001), lactate/albumin (p= 0.007), and CRP/albumin (p=0.001) was higher in deceased patients compared to surviving patients. Prealbumin and albumin were lower in non-survivors (p=0.001). When the factors affecting mortality were examined by multivariate analysis, it was determined that none of the laboratory parameters alone predicted mortality.

SOFA score was the only independent risk factor indicating mortality in the geriatric patient population with sepsis (OR=1.886 (1.410-2.510), p<0.001).

Conclusion

In conclusion, we demonstrated that high age and parameters of nutrition indicators did not affect mortality in geriatric patients hospitalized in the intensive care unit due to sepsis.

In our study, the SOFA score was an independent risk factor affecting mortality in geriatric patients with sepsis, as in all sepsis cases.

## Introduction

Nine percent of the world's population is 65 years and older, and this rate is expected to rise to 22% in the future [1]. The increase in this rate may pose a significant problem in health systems in the future. The majority of patients (42%-52%) treated in intensive care units (ICU) consists of the geriatric patient population [2,3]. One of the most common causes of mortality and morbidity in elderly patients is sepsis. At the same time, sepsis has been defined as the most critical disease of the elderly [4]. The mortality rate due to sepsis increases day by day in patients over 65 years of age [5]. Malnutrition is very common in elderly patients and has been indicated to affect mortality and morbidity [6]. Due to increased gluconeogenesis and increased muscle catabolism in sepsis, more than 0.5 g/kg/day of body protein loss occurs in these patients [7].

While the synthesis rate of proteins made in the liver (i.e., albumin, transferrin, prealbumin levels, retinol-binding protein, and fibronectin) decreases, acute phase protein synthesis accelerates. Tissues characterized by rapidly proliferating cells such as enterocytes, immune cells, granulation tissue, and keratinocytes show early changes when protein synthesis capacity is reduced. Glutamine utilization is accelerated in these tissues [8]. Hypoalbuminemia and prealbumin are also indicators of malnutrition and have long been shown to affect mortality and morbidity in elderly patients. Inflammation significantly affects prealbumin and albumin levels. After excluding inflammation, food intake has been reported to account for only a small part of the variance in prealbumin [6]. An increase of ≥ 2 points in Sequential Organ Failure Assessment (SOFA) in patients with infection predicts hospital mortality at a high rate [9]. Early recognition of elderly sepsis patients at high risk of death can help improve care and reduce costs. Our study aimed to evaluate the clinical features of patients admitted to the tertiary intensive care unit with the diagnosis of sepsis and evaluate the effect of the SOFA score, prealbumin, albumin, and other laboratory parameters on hospital mortality.

## Materials and methods

Geriatric (65 years and older) patients admitted to the tertiary intensive care unit of Kayseri Provincial Training and Research Hospital between August 1, 2021 and April 1, 2022 were determined from the hospital's database. Ethics committee approval of this retrospective study was obtained from the ethics committee of Kayseri City Hospital (No: 685/18.08.2022).

Patients diagnosed with sepsis and septic shock according to the Sepsis-3 consensus were included in the study. Organ dysfunction due to dysregulated host response to infection was diagnosed as sepsis. The task force recommended that an increase in the SOFA score of at least two points should be used when infection was encountered as a criterion of sepsis. The baseline SOFA score may be considered zero in patients without prior organ dysfunction.

Patients with liver failure and active malignancy were excluded since these conditions affect albumin and prealbumin levels. Patients with negative or trace amounts of protein in the complete urinalysis at admission were included. The demographic data of the patients on the first day of their admission to the intensive care unit at the hospitalization were recorded.

Information regarding the age, gender, body mass index (BMI), Glasgow Coma Scale (GCS), SOFA score, Nutrition Risk Screening 2002 (NRS 2002), the time of sepsis/septic shock diagnosis, the date of admission to the intensive care unit, and the potential source of sepsis (pulmonary, urinary tract, CNS, catheter-related blood infection, gastrointestinal system, wound site) were recorded. At the 24th hour of admission to the intensive care unit, the first blood gas and hemogram values, procalcitonin and CRP values, Acute Physiology and Chronic Health Evaluation II (APACHE II), and SOFA scores of the patients were recorded. The culture samples' results were noted on the day of admission to the intensive care unit.

The need for renal replacement therapy and mechanical ventilation, developing complications (ventilator-associated pneumonia, catheter infection, myocardial infarction, GIS bleeding, deep vein thrombosis), and mortality during his stay in the intensive care unit was determined in the data collection form. The diagnosis of acute kidney injury (AKI) was staged according to the Kidney Disease Improving Global Outcomes (KDIGO) criteria at the time of admission [10]. AKI was defined as an increase of x0.3 mg/dL in serum creatinine within 48 hours, or an increase in serum creatinine to x1.5 times baseline known or estimated to have occurred in the previous seven days; or urine volume 0.5 ml/kg/hr for six hours.

Statistics

Clinical records were recorded on a data collection form. The obtained data were transferred to the database for statistical analysis with SPSS Statistics package version 25.0 (Chicago, IL, USA). Shapiro-Wilk test of normality was applied. Categorical variables were presented as numbers (n) and percentages (%). Continuous variable results were expressed as medians and interquartile range values.

Mann-Whitney U test for continuous variables and χ2 or Fisher's precision test for categorical variables were used to detect significant differences between groups. Logistic regression multivariate analysis was performed for factors affecting hospital mortality.

A forward-step binary logistic regression analysis was performed to determine the independent factors on mortality, with the variables determined to have a p-value of <0.1 in univariate analysis, and the results were presented with an odds ratio (OR) and confidence interval values.

## Results

A total of 653 patients admitted to the tertiary internal medicine intensive care unit were evaluated. Three hundred forty (52%) of the patients were geriatric (65 years and over) patients admitted to the intensive care unit due to sepsis and septic shock. Forty-one patients were excluded because of proteinuria, 30 patients due to active malignancy, and 15 patients because of liver failure. A population of 254 geriatric patients was admitted to the intensive care unit due to sepsis and septic shock.

In-hospital mortality occurred in 122 patients. The patients were divided into two groups, survivors and non-survivors, and demographic and clinical characteristics were compared between the two groups (Table 1).

**Table 1:** Demographic and clinical characteristics of elderly patients hospitalized in the tertiary intensive care unit with sepsis according to their surviving conditions BMI; Body Mass Index, CNS; central nervous system, NRS2002; Nutrition Risk Screening 2002, ICU; Intensive Care Unit, CKD; Chronic Kidney Disease, COPD; Chronic Obstructive Pulmonary Disease, CAD; Coronary Artery Disease, SOFA; Sequential Organ Failure Assessment, APACHE II; Acute Physiology and Chronic Health Evaluation, AKI; Acute Kidney Injury, RRT; Renal replacement therapy, VIP; ventilator-associated pneumonia, GIS; gastrointestinal system, DVT; deep vein thrombosis

	Overall (n=254)	Survivors (n=132)	Non-survivors (n=122)	P-value
Mean age median year (interquartile range)	76(70-84)	76(71-84)	76(70-84)	0.896
Gender n (%) Male	204(58.11)	56(42.4)	77(63.1)	0.079
BMI (interquartile range)	24(21-28)	24(21-29)	24(21-27)	0.110
Source of infection; Pulmonary n (%)	81(31.9)	34(25.8)	47(38.5)	0.178
Source of infection; Urinary Tract n (%)	132(52)	80(60.6)	52(42.6)	<0.01
Source of infection; CNS n (%)	2(0.8)	0(0)	2(1.6)	N
Source of infection; Catheter-related blood infection n (%)	7(2.8)	3(2.3)	4(3.3)	0.253
Source of infection; Gastrointestinal system n (%)	27(10.6)	15(11.4)	12(9.8)	0.235
Source of infection; Wound site n (%)	5(2)	0(0)	5(4.1)	N
NRS2002 (interquartile range)	3(1-4)	2(1-2)	2(1-3)	0.857
Decubitis ulcer before ICU admission n (%)	94(37)	37(28)	57(46.7)	0.034
CKD n (%)	47(18.5)	21(15.9)	26(21.3)	0.027
Diabetes mellitus n (%)	81(31.9)	37(28)	44(36.1)	0.004
COPD n (%)	42(16.5)	17(12.9)	25(20.5)	<0.001
CAD n (%)	132(52.8)	59(44.7)	75(61.5)	0.058
Hypertension n (%)	180(70.9)	93(70.5)	87(71.3)	0.613
Dementia n (%)	48(18.9)	18(14.8)	30(22.7)	0.001
Nutritional Support Oral n (%)	113(44.1)	87(65.9)	25(20.5)	<0.001
Nutritional Support; Enteral n (%)	106(41.4)	33(25)	73(59.8)	<0.001
Nutritional Support; Parenteral n (%)	36(14.1)	12(9.1)	57(19.7)	0.002
Glasgow coma scale (interquartile range)	13(11-14)	13(12-14)	12(9-14)	<0.001
SOFA (interquartile range)	3(2-4)	3(2-3)	4(3-6)	<0.001
APACHE II (interquartile range)	23.5(18-28.5)	22(17-27)	24(18-31)	0.046
Invasive Mechanical ventilation n (%)	108(42.5)	3(2.3)	105(86.1)	<0.001
AKI Stage 1 n(%)	34(13.4)	21(15.9)	13(10.7)	0.031
AKI Stage 2 n(%)	24(9.4)	8(6.1)	16(13.1)	0.056
AKI Stage 3 n(%)	93(36.6)	36(27.3)	57(46.7)	0.028
RRT n (%)	93(36.5)	32(24.2)	61(50)	<0.001
Use of vasoactive agent n (%)	132(51.8)	24(18.2)	108(88.5)	<0.001
Complications during ICU stay n (%)	42(16.5)	7(5)	37(30)	0.002
VIP n (%)	32(12.5)	2(1.5)	30(24.6)	<0.001
Catheter Infection n (%)	4(1.6)	2(1.5)	2(1.6)	0.875
Myocardial Infarction n (%)	3(1.2)	1(0.8)	2(1.6)	0.947
GIS bleeding n (%)	2(0.8)	1(0.8)	1(0.8)	0.981
DVT n (%)	1(0.4)	1(0.8)	0(0)	0.002
Median days in ICU (interquartile range)	9(5-14)	7(4-11)	11(7-18)	0.001
Median days in hospital (interquartile range)	15(10-24)	16(11-25)	14(9-23)	0.958

There was no difference in age between the groups (76 (71-84) vs. 76 (70-84), p=0.896). Chronic renal failure (p=0.027), diabetes mellitus (p=0.004), chronic obstructive pulmonary disease (p<0.001), and dementia (p=0.001) were more common in non-survivors than in survivors.

**Figure 1:**
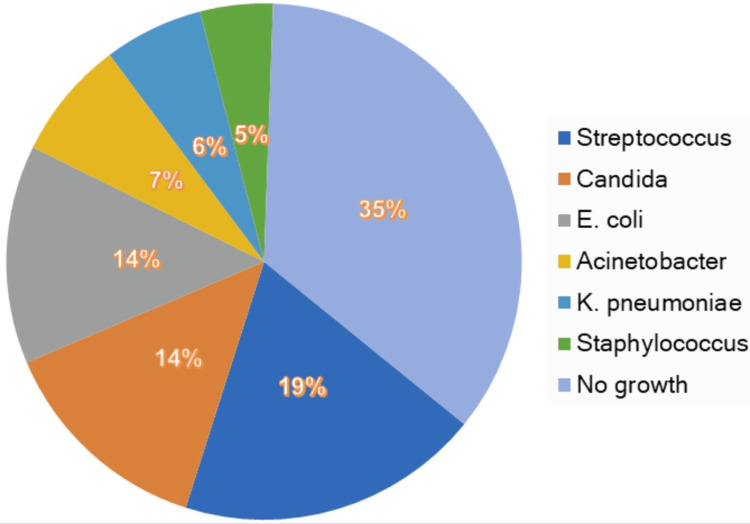
Microorganisms causing sepsis and septic shock E. coli; Escherichia coli, K. pneumonia; Klebsiella pneumoniae

In the non-survivor group, Glasgow coma scale (p<0.001) scores were lower, and SOFA (p<0.001) and APACHE II (p=0.046) scores were higher than survivors. The most frequent focus of infection was urinary tract infections (52%). The microorganisms causing sepsis in the patients were mostly streptococci in 46 patients and Escherichia coli (E. coli) in 36 patients. No microorganism was detected in 85 patients (Figure 1).

 

Complications developed in 42 (16.5%) patients during their stay in the intensive care unit; the most common complication was ventilator-associated pneumonia in 32 (12.5%). Patients who did not survive had more complications than those who survived (37 [30%] vs. 7 [5%]) (p=0.002). The laboratory parameters of the elderly patients with sepsis were compared in Table 2 according to whether they survived.

**Table 2:** Baseline laboratory parameters of elderly patients with sepsis and septic shock GGT; Gamma-glutamyl transferase, ESR; erythrocyte sedimentation rate,TSH; thyroid-stimulating hormone, CRP; C-reactive protein, NLR; neutrophil-to-lymphocyte ratio, RBC; red blood cell, RDW; red cell distribution width, CK; Creatine kinase, CK-MB; myocardial band creatine kinase, INR; international normalized ratio, LDH; lactate dehydrogenase, BUN; blood urea nitrogen

	Overall (n=254)	Survivors (n=132)	Non-survivors (n=122)	P-value
Blood urea nitrogen mg/dL	40(26-66)	37(22-64)	49(30-66)	0.013
Creatinine mg/dL	1.61(0.91-3.39)	1.33(0.84-3.30)	1.7(1-3.4)	0.073
Aspartate aminotransferase IU/L	25(18-49)	23(17-38)	28(18-52)	0.038
Alanine aminotransferase IU/L	16(8-29)	15(8-29)	17(8.75-29)	0.648
Lactate Dehydrogenase U/L	284(228-379)	272(223-347)	316(237-424)	0.014
Total bilirubin mg/dL	0.6(0.4-1.1)	0.6(0.4-0.9)	0.7(0.4-1.6)	0.070
GGT U/L	33.5(16.75-95.25)	29(15-68)	38(19-117)	0.043
Sodium mmol/L	138(133-142)	138(133-141)	137(130-140)	0.778
Potassium mmol/L	4.3(3.7-5.1)	4.2(3.7-5.2)	4.35(3.80-5)	0.675
Phosphor mg/dL	3.8(3-4.8)	3.5(2.8-4.5)	4(3.25-5.25)	0.002
Magnesium mg/dL	1.95(1.71-2.27)	1.9(1.71-2.26)	1.96(1.70-2.31)	0.499
Calcium mg/dL	8.1(7.7-8.6)	8.2(7.8-8.6)	8(7.55-8.5)	0.019
Albumin g/L	28(24-32)	30(27-33)	27(23-31)	0.001
Prealbumin g/L	0.08(0.03-0.13)	0.11(0.05-0.170)	0.07(0.02-0.12)	0.001
Ferritin µg/L	411(144-1010)	285(104-686)	609(232-1385)	<0.001
Transferrin saturation	18(10-31)	18(10-26)	20(10-36)	0.378
B12 ng/L	505(346-981)	449(308-781)	629(417-1071)	<0.001
Folic acid µg/L	5.1(3.3-8.93)	5.1(3.5-9.6)	4.5(2.90-8.20)	0.178
ESR mm/h	35(18-56)	32(18-52)	38(17-60)	0.154
sT_3 _ng/L	1.5(1.2-2.1)	1.5(1.2-2)	1.5(1.10-1.90)	0.409
ST_4_ ng/L	11.6(9.5-14.05)	12.05(9.9-14.3)	11.35(8.80-13.42)	0.040
TSH ng/L	1.2(0.67-2.49)	1.23(0.66-2.07)	1.15(0.67-3.077)	0.764
CRP mg/L	80(27-177)	72(20-149)	93(33-196)	0.027
Procalcitonin µg/L	0.58(0.19-2.49)	0.39(0.16-1.72)	0.62(0.48-2.61)	0.002
White blood cell /mm^3^	9670(6275-13557)	9365(6612-13060)	10160(5967-13932)	0:402
Neutrophil /mm^3^	7675(4830-11557)	7496(5162-11205)	8200(4517-12315)	0.447
Lymphocyte /mm^3^	975(547-1445)	1110(622-1580)	835(480-1357)	0.015
NLR	7.33(4.06-15.86)	6.82(3.75-13.47)	8.43(4.06-16.21)	0.050
Hemoglobin g/dL	10(8.67-11.80)	10.40(8.8-11.6)	9.7(8.5-12.12)	0.528
RBC 10^6^/µL	3.63(3.02-4.18)	3.78(3.19-4.15)	3.43(2.97-4.26)	0.207
RDW %	16(14.6-18.40)	15.35(14.22-17.77)	16.5(15.4-18.82)	0.001
Platelets /mm^3^	203000(127750-276000)	224000(146000-272250)	187000(108500-293250)	0.181
CK U/L	64(38-165)	57(38-151)	70(35-208)	0.370
CK-MB U/L	26(17-37)	23(16-35)	29(20-42)	0.004
hs Troponin T ng/L	59(30-101)	44(26-92)	70(40-125)	0.002
INR	1.28(1.15-1.45)	1.23(1.12-1.40)	1.35(1.18-1.57)	0.002
Fibrinogen mg/L	4720(3145-5742)	4655(3350-5552)	4870(3140-6110)	0.559
D dimer µg/L	4087(1762-8567)	3796(1765-8555)	4710(1728-8679)	0.405
Lactate mmol/L	1.7(1.2-2.92)	1.5(0.9-1.8)	1.9(1.3-3.25)	0.006
LDH/albumin	10.51(8-14.08)	9.631(7.468-12.044)	11.75(8.88-16.80)	<0.001
Lactate/albumin	0.06(0.04-0.10)	0.05(0.035-0.091)	0.07(0.04-0.12)	<0.001
CRP/albumin	2.79(0.83-6.34)	2.47(0.658-5.478)	3.41(1.41-7.10	0.007
BUN/albumin	1.44(0.93-2.31)	1.224(0.735-2.078)	1.75(1.12-2.61)	0.001

BUN (p=0.013), LDH (p=0.014), LDH/albumin (p <0.001), BUN/albumin (p <0.001), lactate/albumin (p=0.007), and CRP/albumin were higher in non-surviving patients compared to survivors (p=0.001). Prealbumin and albumin levels were lower in non-survivors (p=0.001). When the factors affecting mortality were examined by multivariate analysis, it was determined that none of the laboratory parameters alone predicted mortality. SOFA score was the only independent risk factor affecting mortality in the geriatric patient population with sepsis (OR=1.886 [1.410-2.510], p<0.001) (Table 3).

**Table 3:** Risk factors for mortality in elderly critically ill patients with sepsis and septic shock S.E.; standard error, O.R; odds ratio, C.I; confidence intervals

	B	S.E.	p	OR	95% C.I.for Exp(B)	
Variables					Lower	Upper
SOFA	0.635	0.149	<0.001	1.886	1.410	2.510
APACHE II	-0.016	0.035	0.638	0.984	0.919	1.054
LDH	0.000	0.002	0.822	1.000	0.997	1.004
Albumin	-0.066.	0.053	0.213	0.936	0.843	1.039
Prealbumin	-2.862	2.503	0.942	0.999	0.985	1.014
BUN	0.000	0.033	0.995	1.000	0.938	1.054
LDH/Albumin	0.016	0.056	0.783	1.016	0.909	1.134
BUN/albumin	0.033	0.182	0.855	1.034	0.724	1.477
Lactate/albumin	1.266	2.567	0.622	3.547	0.023	543.121
CRP/Albumin	-0.032	0.195	0.871	0.969	0.661	1.421

## Discussion

The need for intensive care is increasing day by day, and most of the patient population requiring it is geriatric patients. Infection is an independent risk factor for a higher mortality rate in older patients [11]. Martin et al. indicated that the incidence of sepsis is higher in geriatric patients than in non-geriatric patients [12].

In our study, 70.8% of the patients hospitalized in the intensive care unit were geriatric patients with sepsis and septic shock, and the mortality rate was 48%. The only independent risk factor affecting mortality in these patients was the SOFA score. Moreover, age was not a risk factor affecting mortality. The average life span in Turkey is about 77 years. If the study population included patients aged 65 and above, that could explain the no difference between the two groups as few people exist who are >77. Our findings have revealed how frequent and critical sepsis is in geriatric patients.

In the study of Gorgulu et al., 69.2% of the patients hospitalized in the intensive care unit with sepsis consisted of the geriatric patient population, and the mortality rate was 77% [13]. In another study, all sepsis patients admitted to the intensive care unit were divided into three groups under 60 years, 60-80 years old, and over 80 years old, and the mortality rates were 45.6%, 60.7 and 78.9%, respectively [14]. Lower mortality might be due to early diagnosis and treatment of sepsis and early diagnosis and treatment of the patients according to the Sepsis-3 definitions in our study. The SOFA score is used for the Sepsis-3 definition.

In another study performed recently in geriatric patients with sepsis and septic shock, in line with our findings, age did not affect mortality, while the SOFA score indicating organ failure was an independent risk factor determining mortality [15]. The most common foci of infection in our patients were urinary infections and pneumonia. Studies have demonstrated that pneumonia and urinary tract infections are the most common source of infection and gram-negative bacteria [12,14,16].

Hypoalbuminemia is observed with muscle wasting in geriatric patients and affects morbidity and mortality [17]. Since muscle wasting due to malnutrition is expected in the geriatric patient population, we first evaluated its relationship with mortality in the intensive care unit, predicting that albumin, prealbumin, albumin-based ratios, and NRS2002 score, which indicate malnutrition, affect mortality. We determined a higher mortality rate in patients with low albumin and prealbumin levels in intensive care unit admission, but it did not affect mortality in multivariate analyses. In a study performed on the geriatric patient population, the predictive value of albumin and prealbumin levels on mortality was 78%. Still, comorbidities affecting albumin and prealbumin values were not excluded in this study [6].

In a recent study, albumin and CRP were indicated as risk factors that independently affect 28-day mortality in sepsis patients over 65 years of age (p<.001). However, the sensitivity and specificity of the SOFA score were not higher in demonstrating mortality [18]. The results of our study were different may be the presence of proteinuria in our exclusion criteria, which may have affected our results. However, the importance of the SOFA score in predicting mortality is obvious.

Limitations

This research was a retrospective observational study, and patients were included in the study according to the data in the files. The study results may have been affected as there may be missing data. Our sample size, though statistically sufficient, was small. More accurate results can be obtained with larger sample size and prospective studies.

## Conclusions

In conclusion, we showed in this study that patients with sepsis and septic shock admitted to the intensive care unit have a high incidence and mortality. When the independent risk factors affecting mortality in these patients were examined, it was seen that age did not affect it. At the same time, prealbumin and albumin, which are used as nutritional indicators, could not be shown to affect mortality either. However, the SOFA score was an independent risk factor affecting mortality in geriatric patients with sepsis, as in all sepsis cases. Larger randomized studies are needed for biomarkers that predict mortality in sepsis and septic shock in elderly patients.
